# Synthesis of three-dimensional porous hyper-crosslinked polymers via thiol–yne reaction

**DOI:** 10.3762/bjoc.12.252

**Published:** 2016-11-29

**Authors:** Mathias Lang, Alexandra Schade, Stefan Bräse

**Affiliations:** 1 Institute of Organic Chemistry, Karlsruhe Institute of Technology – Campus South, Fritz-Haber-Weg 6, 76131 Karlsruhe, Germany; 2 Institute of Toxicology and Genetics, Karlsruhe Institute of Technology – Campus North, Hermann-von-Helmholtz-Platz 1, 76344 Eggenstein-Leopoldshafen, Germany

**Keywords:** three-dimensional, porous hyper-crosslinked polymers, thiol–yne

## Abstract

Herein we report the syntheses of two porous hyper-crosslinked polymers (HCPs) via thiol–yne reaction with rigid tetrahedral and *pseudo*-octahedral core structures. Sorption measurements with nitrogen gas at 77 K revealed BET-surface areas up to 650 m²/g. Those networks also showed a high thermal stability as well as insolubility in common organic solvents.

## Introduction

The synthesis of different organic networks has been previously reported. Among them, especially tetraphenylmethane cores are widely employed in the synthesis of covalent organic frameworks (COFs) [1–2], porous aromatic frameworks (PAFs) [3], porous polymer networks (PPNs) [4] and hyper-crosslinked polymers (HCPs) [5]. These organic networks are, due to their large surface areas, of interest in gas storage [6], gas separation [7] and catalysis [8–10]. For the synthesis of organic networks, many different reaction types such as condensation reactions [11–12], coupling reactions [3] and click reactions [5,13] have been reported. Herein we present the synthesis of porous, three-dimensional tetraphenylmethane-based networks by another click reaction, the thiol–yne reaction [14–19]. This reaction type has been known for several decades and relived a renaissance in the past decade, especially in material sciences [20–32], due to its mild, and metal-free reaction conditions, high yields and easy purification.

## Results and Discussion

**Scheme 1:**
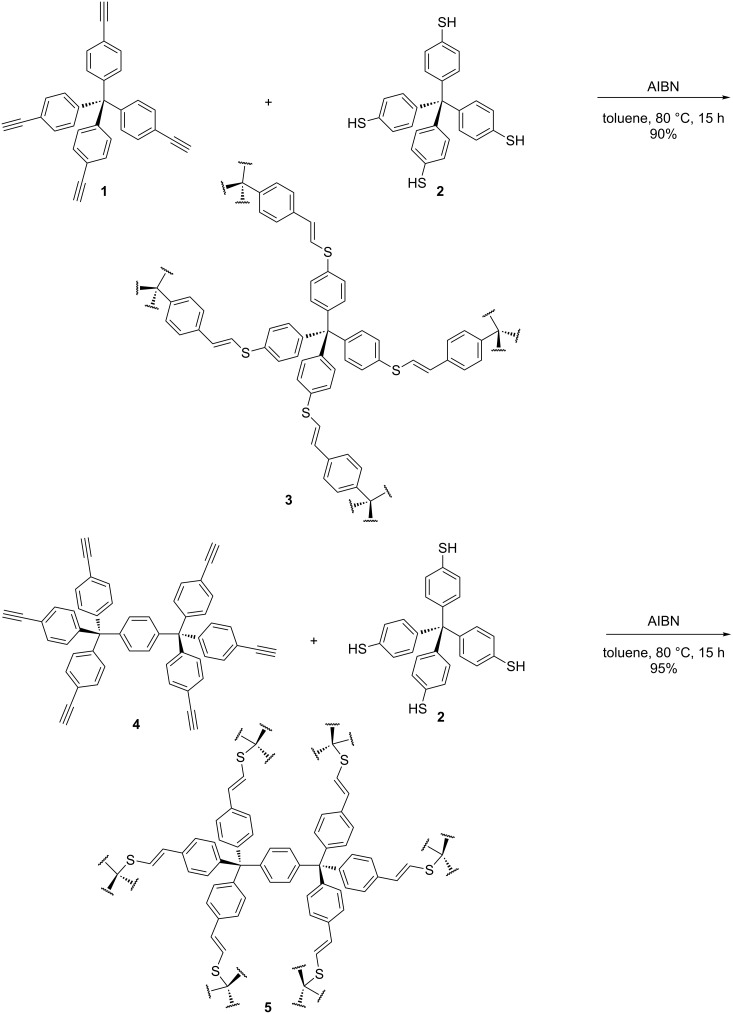
Syntheses of the HCPs **3** and **5** via thiol–yne reaction.

The first network shown here was synthesized by crosslinking the two tetrahedral tetraphenylmethane core structures **1** and **2** via the radical-mediated thiol–yne reaction using AIBN as initiator. The second network was synthesized with tetraphenylmethane core **2** and the *pseudo*-octahedral bistritylbenzene core **4** under the same reaction conditions (Scheme 1). The resulting HCPs **3** and **5** were obtained in 90% and 95% yields, respectively. Both HCPs showed complete insolubility in common organic solvents. The monomers **1** [13], **2** [33] and **4** [34] were synthesized according to literature procedures.

**Figure 1:**
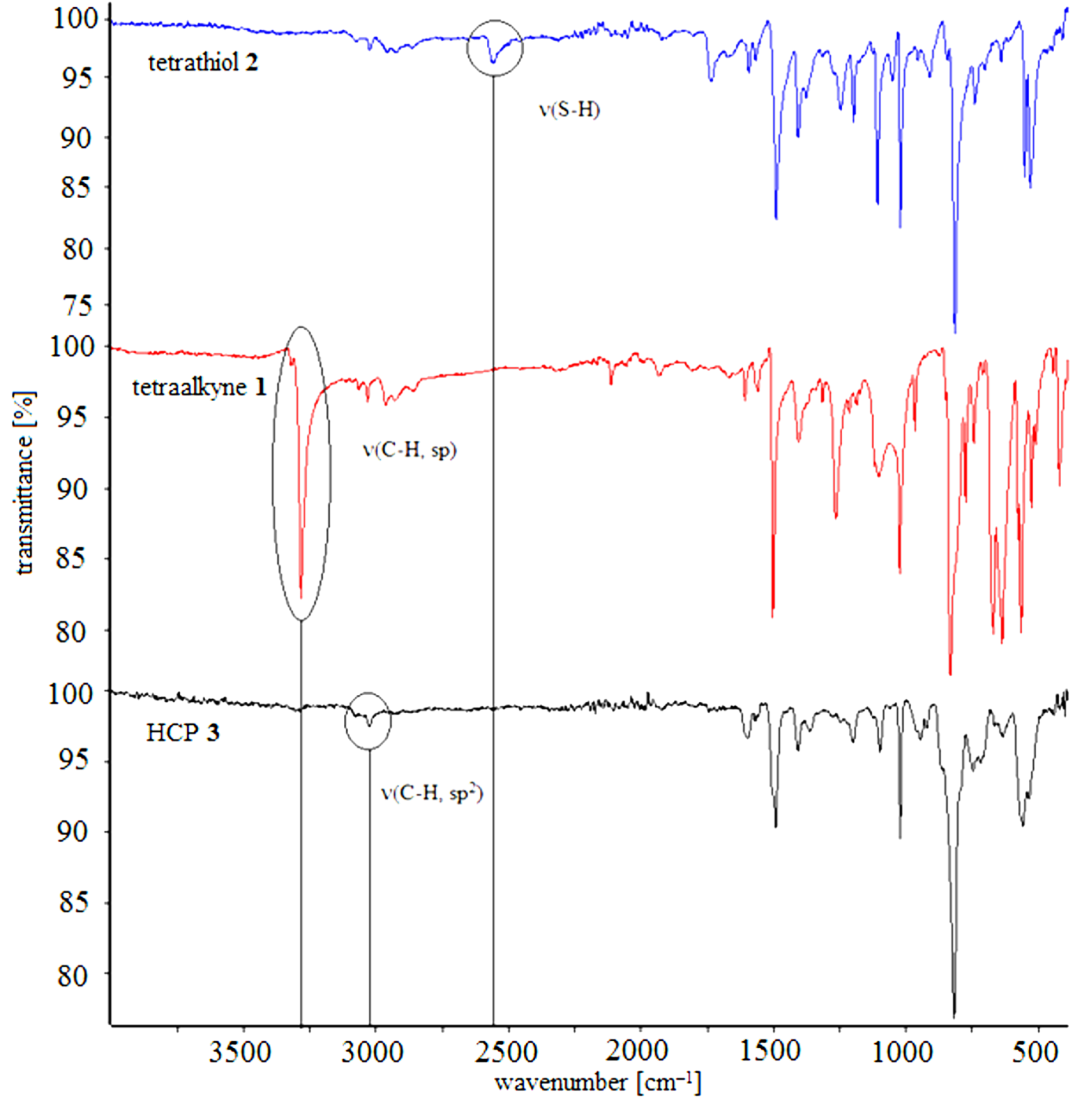
IR-spectra of tetrathiol **2** (blue), tetraalkyne **1** (red) and HCP **3** (black).

**Figure 2:**
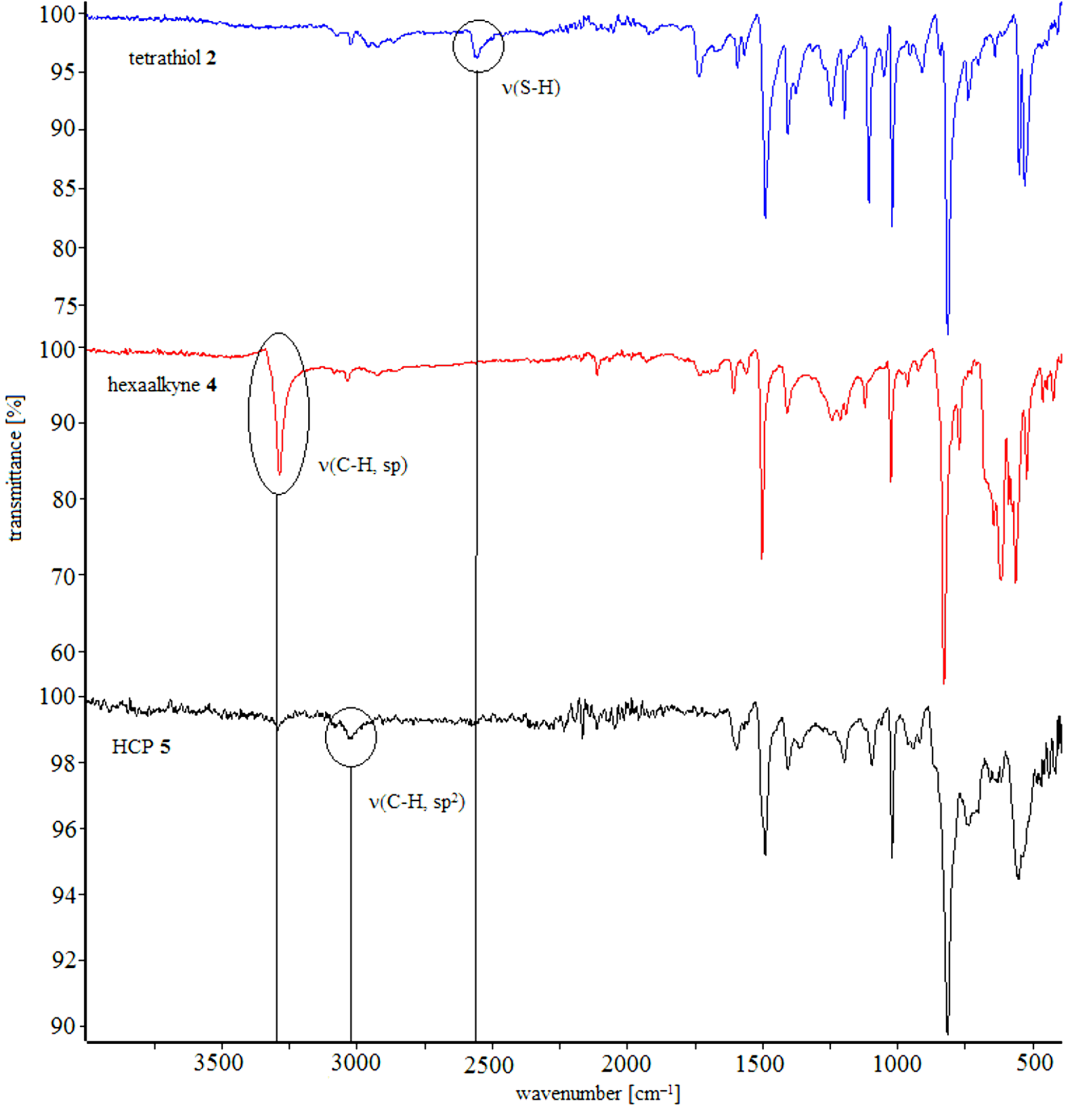
IR-spectra of tetrathiol **2** (blue), hexaalkyne **4** (red) and HCP **3** (black).

The structures of HCPs **3** and **5** were analysed by elemental analysis and IR spectroscopy. The IR spectra of the monomers show the characteristic vibration bands of alkynes at 3300 cm^−1^ and the vibration band at 2550 cm^−1^ of thiols, respectively. However, these characteristic bands are nearly extinguished in the IR spectra of the HCPs showing a high ratio of crosslinking for these reactions. In addition, the vibration bands of the HCPs at 3000 cm^−1^ correspond to the presence of olefinic bonds, which is in accordance to a monoaddition of a thiol to an alkyne. The absence of a vibration band at 2900 cm^−1^ reveals that there are no saturated fragments in the HCPs, again showing that only a monoaddition and no further addition to the corresponding thioacetal or 1,2-disulfide took place (Figure 1 and Figure 2).

The elemental analyses (Table 1) of networks **3** and **5** showed equimolar turnover regarding the number of functional groups of the monomers. Further, TGA measurements showed a high thermal stability of the HCPs. The TGA curves of HCP **3** and **5** are shown in Supporting Information File 1, Figures S5 and S6.

**Table 1:** Elemental analyses of HCPs **3** and **5**.

		C	H	S

HCP **3**	calcd.	80.52	4.66	14.84
	found	79.06	4.71	14.55
HCP **5**	calcd.	81.38	4.68	13.94
	found	79.34	4.49	13.20

**Figure 3:**
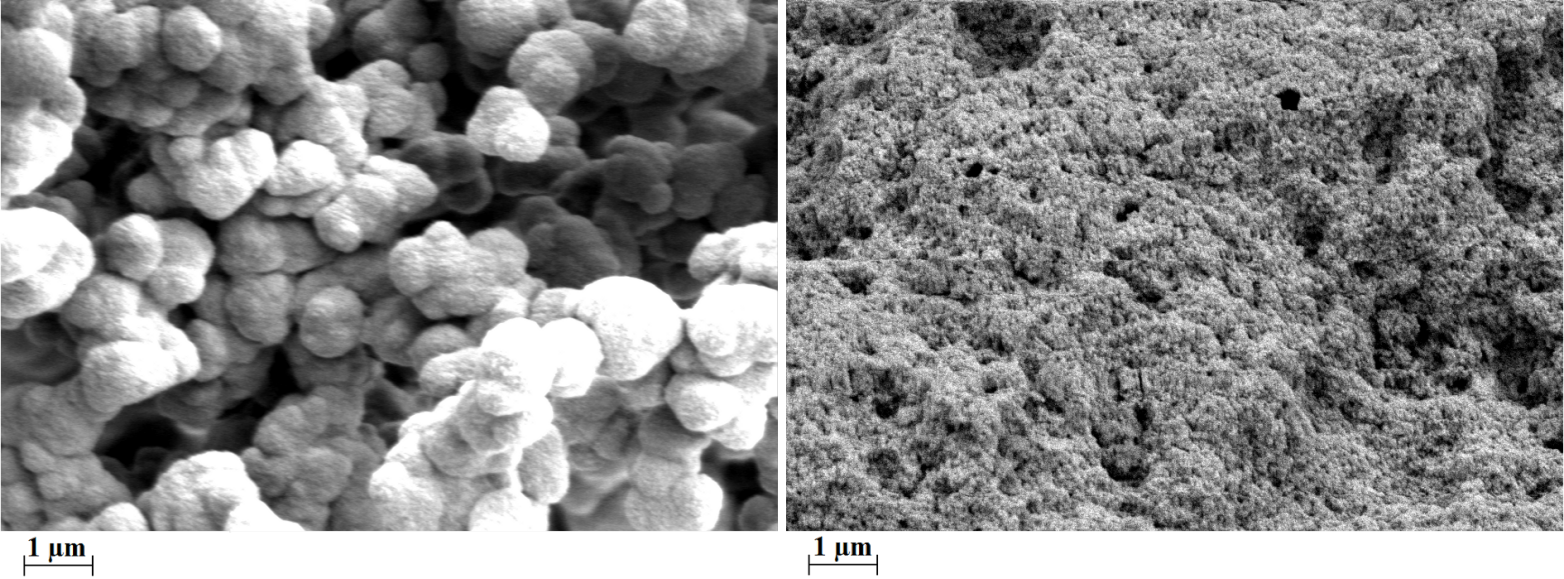
SEM images of HCP **3** (left) and HCP **5** (right).

The SEM pictures (Figure 3) show the amorphous character of the HCPs, which is consistent with the PXRD measurements (see Supporting Information File 1, Figures S3 and S4). The SEM pictures also reveal that HCP **3** consists of particles in the micrometer range while the particle size of HCP **5** is in the sub-micrometre area.

**Figure 4:**
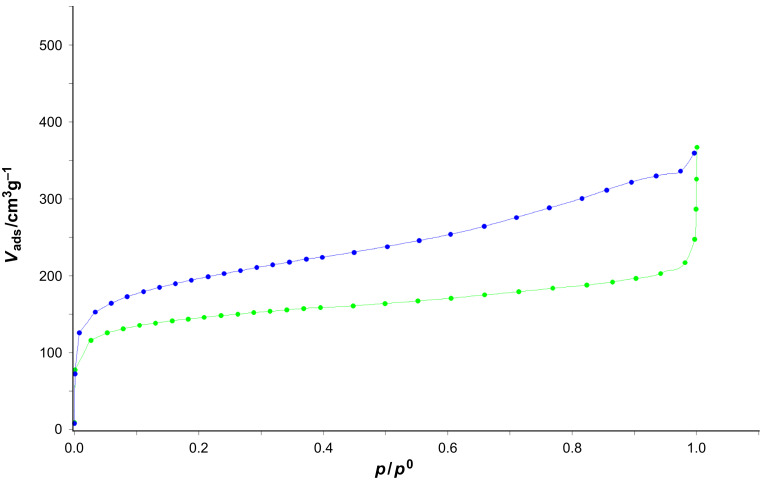
Adsorption isotherms of HCP **3** (green) and HCP **5** (blue) with nitrogen at 77 K. Desorption isotherms are not shown for the sake of improved clarity; they can be viewed in Supporting Information File 1, Figures S1 and S2.

Furthermore, adsorption measurements of HCPs **3** and **5** were carried out with nitrogen gas at 77 K after pre-drying for 16 h at 80 °C in vacuum. Both HCPs showed BET-surface areas >400 m^2^/g and the values of the specific surface area and cumulative volumes of the HCPs are collected in Table 2. The corresponding adsorption isotherms are depicted in Figure 4. The strong slope of the isotherms at low relative pressures indicates a permanent porous character of the materials. Also the step around *p*/*p*^0^ = 0.5 at desorption isotherms (Supporting Information File 1, Figures S1 and S2) indicates the mesoporous character of HCPs **3** and **5**. The low-pressure hysteresis is most probable due to swelling effects or ill-connected pores. The pore-size distributions of HCPs **3** and **5** both show a broad distribution in the microporous scale as well as in the mesoporous scale. These findings also point out that both HCPs have amorphous character as the networks are built up by an irreversible reaction leading to kinetically controlled networks with different sizes of the pores, which is in accordance with the PXRD and SEM measurements stated above. The pore-size distributions of HCPs **3** and **5** are illustrated in Supporting Information File 1 (Figure S8 and S9, respectively).

**Table 2:** Data of adsorption measurements of HCPs **3** and **5**.

HCP	Specific surface area^a^ (BET) [m²/g]	Specific surface area^a^ (Langmuir) [m²/g]	Cumulative volume^b^ [cm³/g]

**3**	470	696	0.314
**5**	650	989	0.510

^a^Surface areas were calculated at a relative pressure range of *p*/*p*^0^ = 0.05–0.3. ^b^Cumulative volumes were calculated at a relative pressure of *p*/*p*^0^ = 0.35–0.95 using the Horvat & Kavazoe method.

## Conclusion

Herein we synthesised amorphous porous tetraphenylmethane-based organic hyper-crosslinked polymers (HCPs) through the thiol–yne reaction. The use of this versatile method reveals advantages such as high yields, cost effectiveness and metal-free crosslinking reaction conditions. The obtained HCPs showed BET surface areas up to 650 m²/g and are insoluble in common organic solvents. The characterisation of the networks was performed using IR spectroscopy, elemental analysis, thermogravimetric analysis (TGA), scanning electron microscopy (SEM), powder X-ray diffraction (PXRD) and adsorption measurements using nitrogen at 77 K.

## Supporting Information

File 1 Experimental procedures and additional measurements.
