# Supplementary material for: Synthesis of three-dimensional porous hyper-crosslinked polymers via thiol–yne reaction
Source: Beilstein J Org Chem. 2016 Nov 29;12:2570–6. doi: 10.3762/bjoc.12.252 (PMC5238528; doi:10.3762/bjoc.12.252)
Supplement: File 1 — Experimental procedures and additional measurements. [file Beilstein_J_Org_Chem-12-2570-s001.pdf]

Supporting Information

for

Synthesis of three-dimensional porous hyper-crosslinked polymers via thiol-yne reaction

Mathias Lang[1], Alexandra Schade[1] and Stefan Bräse[*1,2]

Address: [1]Institute of Organic Chemistry, Karlsruhe Institute of Technology – Campus South, Fritz-Haber-Weg 6, 76131 Karlsruhe, Germany and [2]Institute of Toxicology and Genetics, Karlsruhe Institute of Technology – Campus North, Hermann-von-Helmholtz-Platz 1, 76344 Eggenstein-Leopoldshafen, Germany

Email: Stefan Bräse - bräse@kit.edu

* Corresponding author

Experimental procedures and additional measurements

Contents

General Remarks S2

Synthetic procedures S3

Adsorption measurements S5

PXRD patterns S6

TGA measurements S7

Pore size distributions S9

References S10

General remarks

IR spectra were recorded on a Bruker Alpha spectrometer using the attenuated total reflection technique (ATR). The absorption band is given in wave numbers $\tilde{\nu}$ in cm^{-1}. The intensities of the bands were characterized as follows: vs = very strong 0–10% T, s = strong 11–40% T, m = medium 41–70% T, w = weak 71–90% T, vw = very weak, 91–100% T. Dinitrogen (N_2) adsorption experiments were carried out on a Thermo Scientific™ Surfer at 77 K. Specific surface areas (BET and Langmuir) were calculated at a relative pressure range of p/p_0 = 0.05–0.3. Cumulative volumes were calculated at a relative pressure of p/p_0 = 0.99. Elemental analyses were performed on an Elementar vario MICRO device using a Sartorius M2P precision balance. The following abbreviations were used: calcd. = calculated data, found = measured data. Thermogravimetric analyses (TGA) were performed under N_2 on a Shimadzu TGA-50 thermogravimetric analyser with a heat rate of 10 °C min^{-1}. Powder X-ray diffraction (PXRD) patterns were measured at room temperature using a STORE STADI-P diffractometer with CuKα radiation. Scanning electron microscopy was carried out on a Zeiss Supra 55 and a Zeiss Merlin. Solvents, reagents and chemicals were purchased from Sigma–Aldrich, ABCR and Fisher Scientific. All solvents, reagents and chemicals were used as purchased unless stated otherwise. Tetrakis(4-ethynylphenyl)methane [1], tetrakis(4-mercaptophenyl)methane [2] and 1,4-bis(tris(4'-ethynyl-phenyl)methyl)benzene [3] were synthesized according to literature procedures.

Synthetic procedures

Synthesis of hyper-crosslinked polymers (HCPs) via thiol–yne reaction:

HCP **3**:

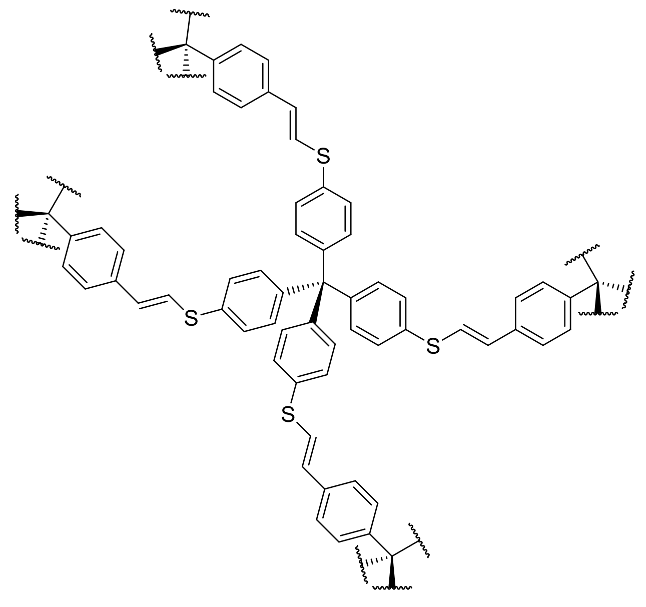

In a sealed reaction vessel, 70.0 mg of tetrakis(4-ethynylphenyl)methane (168 µmol, 1.00 equiv), 75.4 mg of tetrakis(4-mercaptophenyl)methane (168 µmol, 1.00 equiv) and 1.10 mg of AIBN (6.72 µmol, 4.00 mol %) were dissolved in 4 mL toluene and stirred for 15 h at 80 °C. The evolved solid was filtered and washed with toluene (50 mL), ethyl acetate (50 mL), methanol (50 mL), dichloromethane (50 mL) and acetone (50 mL). Finally, the product was dried at 80 °C in vacuo for 16 h to yield 135 mg (90%) of HCP **3** as a yellowish powder. – IR (ATR) $\tilde{\nu}$ (cm^{-1}) = 3023 (vw), 1590 (vw), 1485 (w), 1401 (vw), 1192 (vw), 1091 (vw), 1014 (w), 937 (w), 811 (w), 740 (vw), 625 (vw), 551 (vw). – $C_{58}H_{40}S_4$ (864.2): calc. C 80.52, H 4.66, S 14.84, found C 79.06, H 4.71, S 14.55.

HCP **5**:

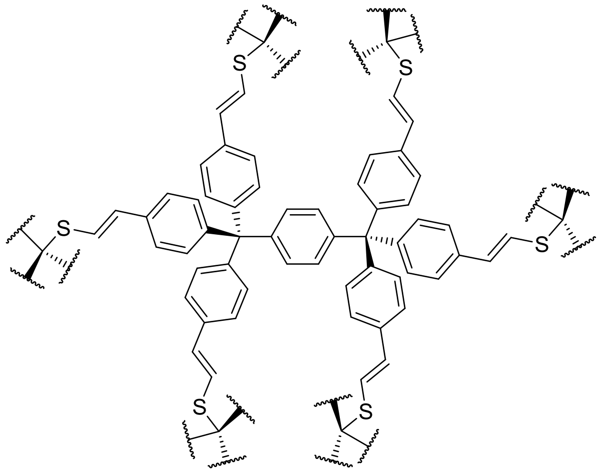

In a sealed reaction vessel, 72.0 mg of 1,4-bis(tris(4'-ethynylphenyl)methyl)benzene (102 µmol, 1.00 equiv), 68.6 mg of tetrakis(4-mercaptophenyl)methane (153 µmol, 1.50 equiv) and 0.70 mg of AIBN (4.07 µmol, 4.00 mol %) were dissolved in 4 mL toluene and stirred for 15 h at 80 °C. The evolved solid was filtered and washed with toluene (50 mL), ethyl acetate (50 mL), methanol (50 mL), dichloromethane (50 mL) and acetone (50 mL). Finally the product was dried at 80 °C in vacuo for 16 h to yield 134 mg (95%) of HCP **5** as an orange powder. – IR (ATR) $\tilde{\nu}$ (cm^{-1}) = 3016 (vw), 1589 (vw), 1484 (vw), 1399 (vw), 1191 (vw), 1089 (vw), 1014 (vw), 936 (vw), 808 (w), 729 (vw), 545 (vw). – $C_{374}H_{256}S_{24}$ (5513.3): calc. C 81.38, H 4.68, S 13.94, found C 79.34, H 4.49, S 13.20.

Adsorption measurements

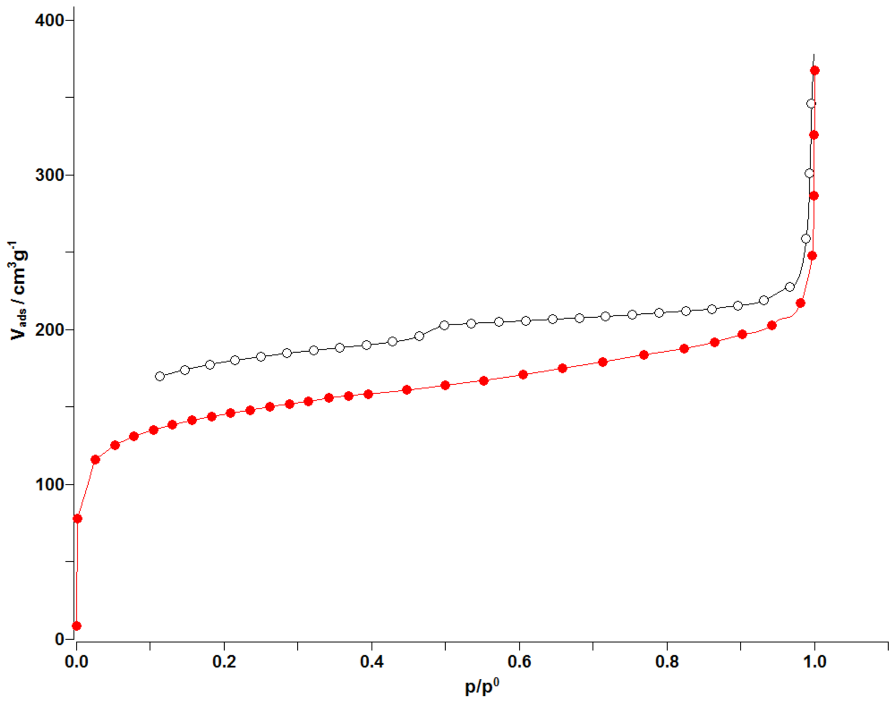

Figure S1: The N_2 isotherms of HCP **3** (red: adsorption, black: desorption) at 77 K (specific surface area (BET) of 470 m²/g).

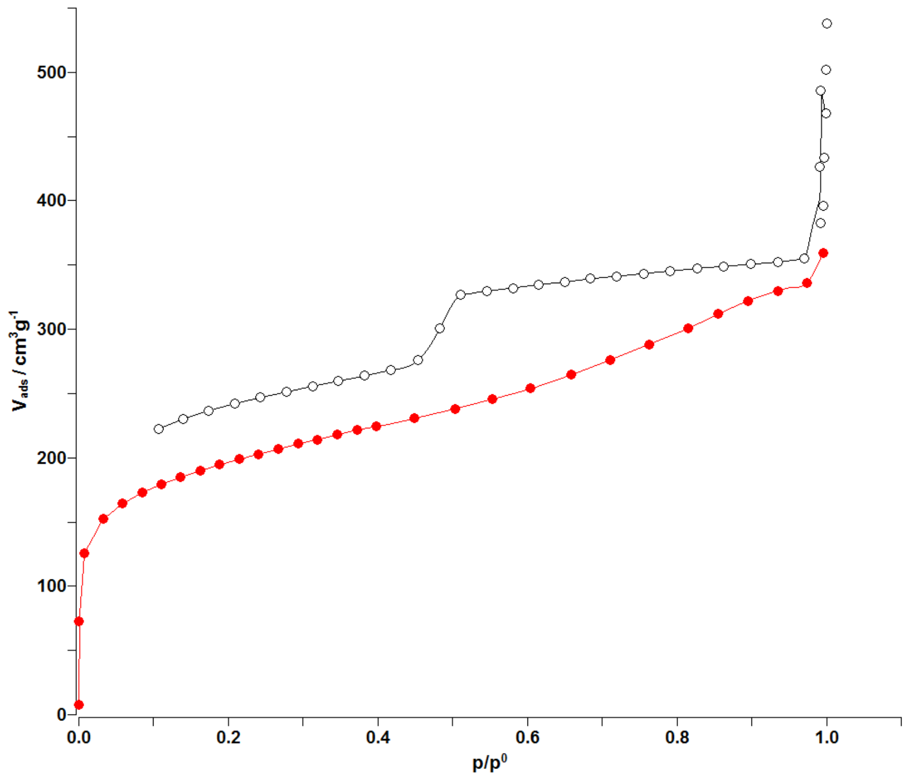

Figure S2: The N_2 isotherms of HCP **5** (red: adsorption, black: desorption) at 77 K (specific surface area (BET) of 650 m²/g).

PXRD patterns

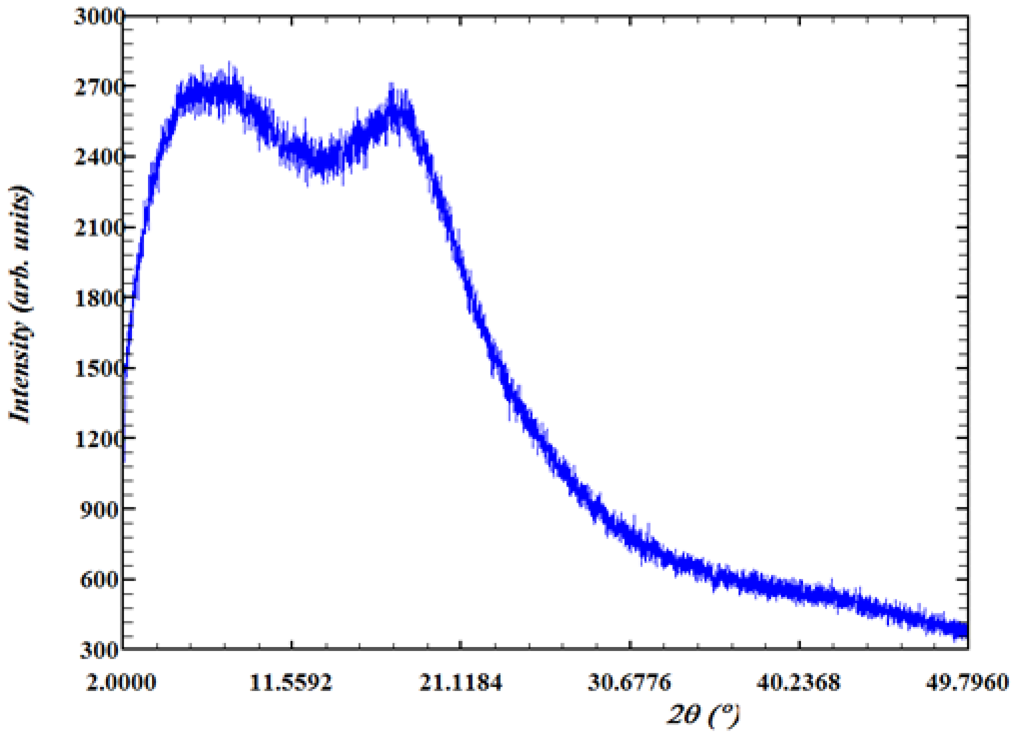

Figure S3: PXRD of HCP **3**.

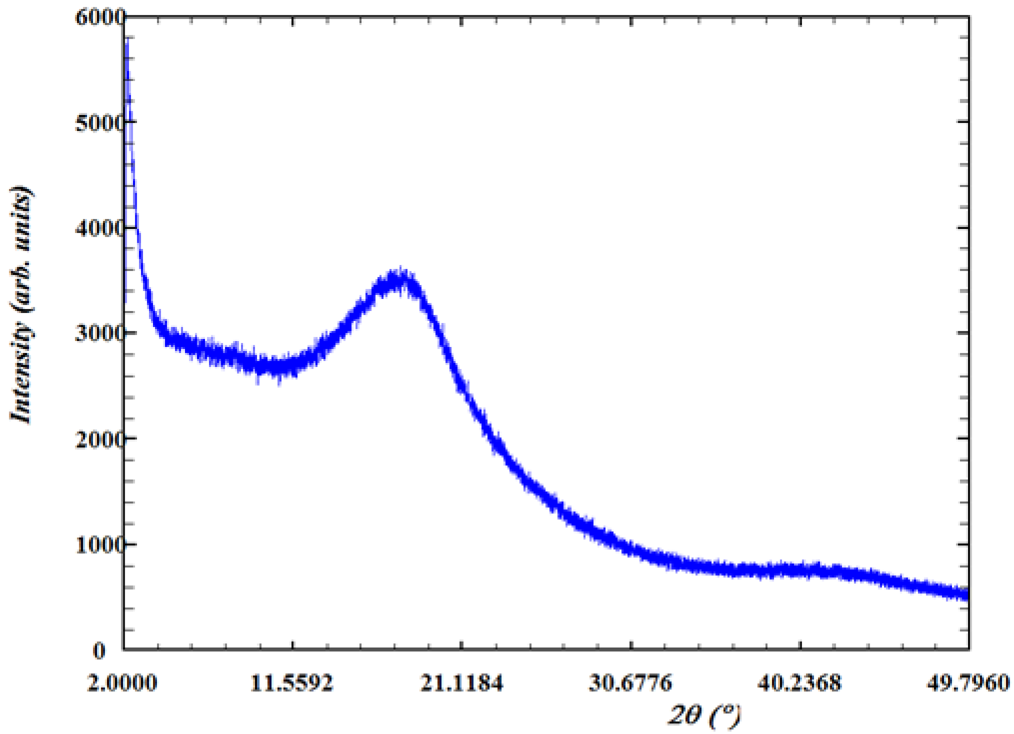

Figure S4: PXRD of HCP **5**.

TGA measurements

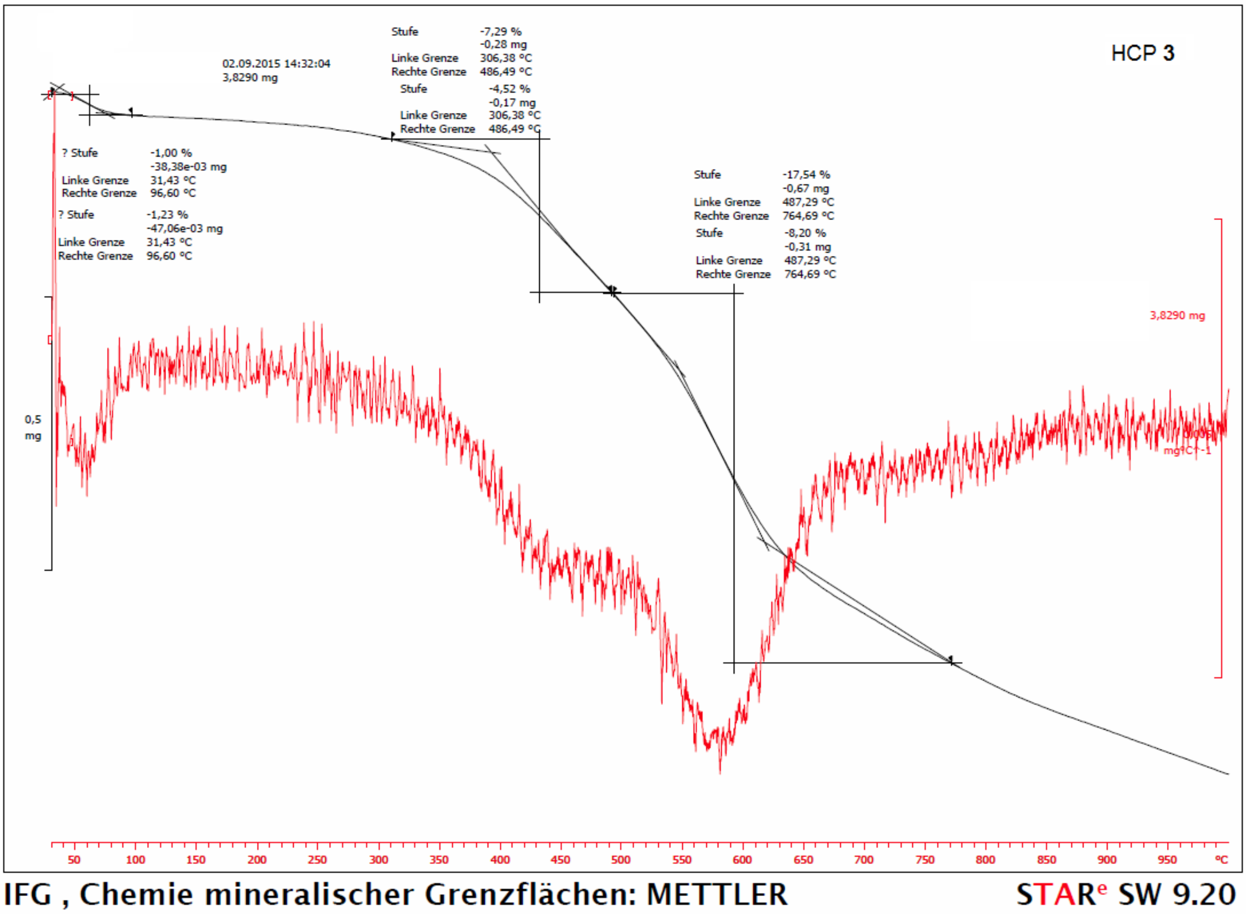

Figure S5: TGA curve of HCP **3**.

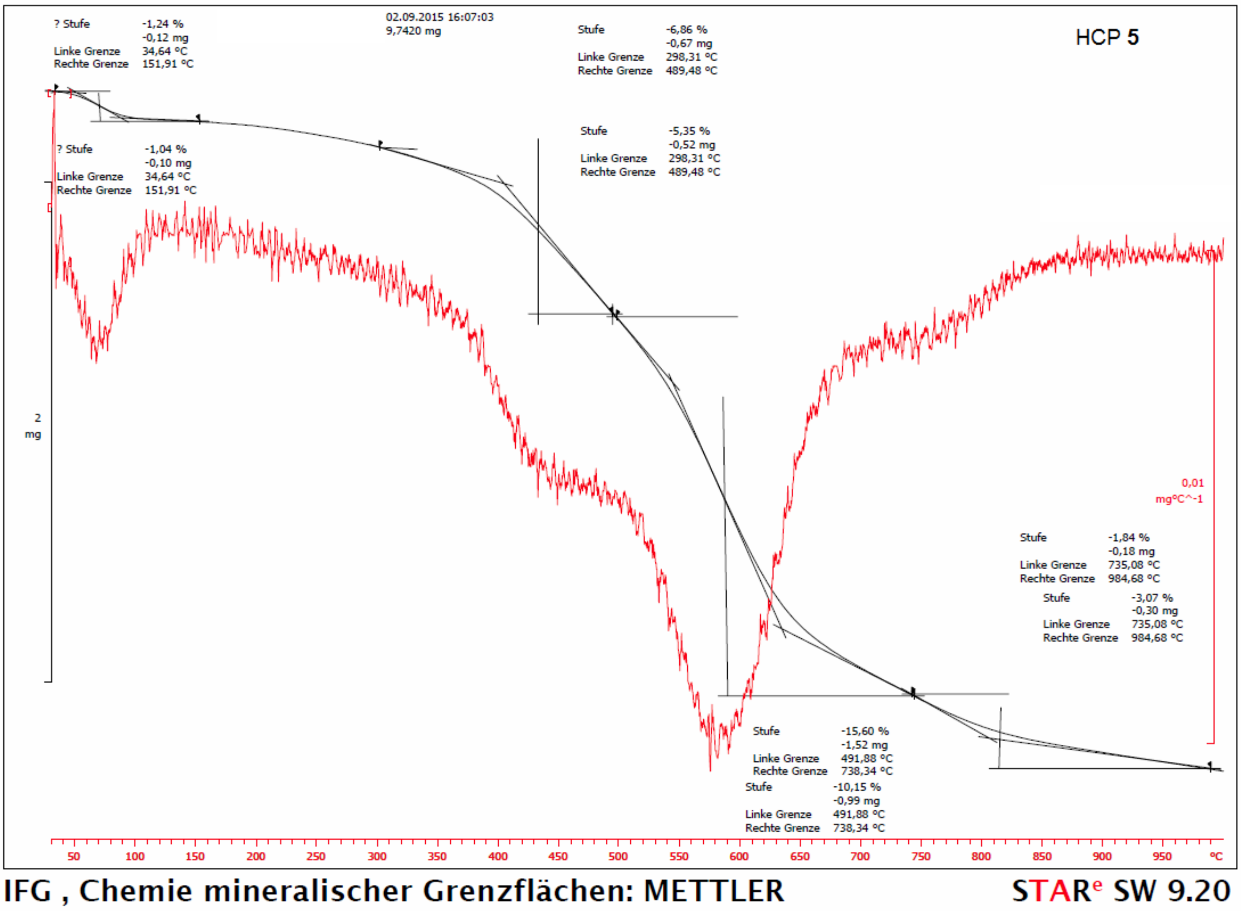

Figure S6: TGA curve of HCP **5**.

Pore size distributions

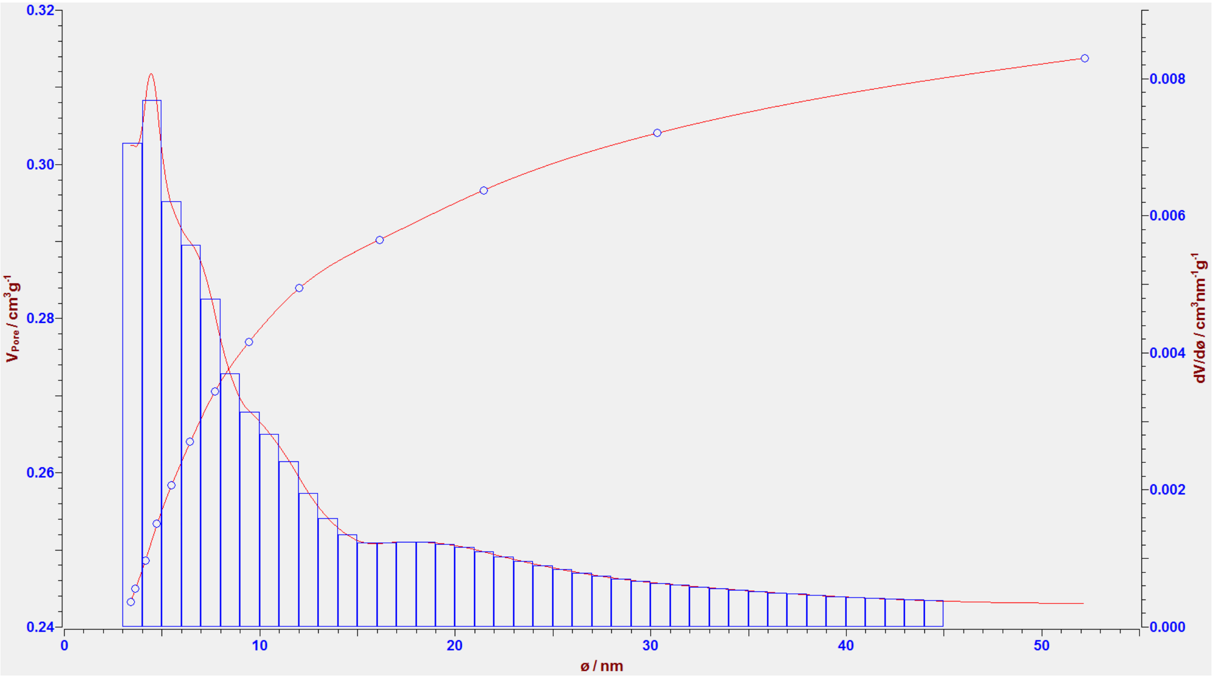

Figure S7: Pore size distribution of HCP **3** using the Horvath and Kawazoe model in the pressure range of p/p_0 = 0.35–0.95.

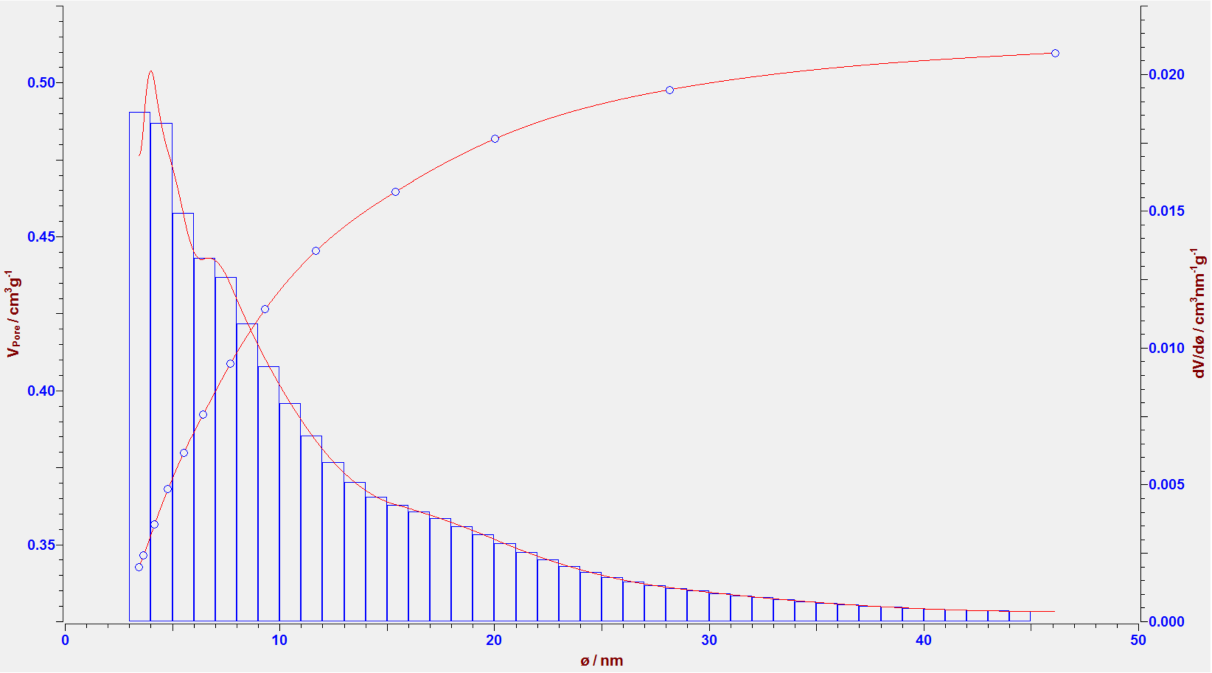

Figure S8: Pore size distribution of HCP **5** using the Horvath and Kawazoe model in the pressure range of p/p_0 = 0.35–0.95.

References

1. Pandey, P.; Farha, O. K.; Spokoyny, A. M.; Mirkin, C. A.; Kanatzidis, M. G.; Hupp, J. T.; Nguyen, S. T. *J. Mater. Chem.* **2011**, *21*, 1700–1703.
2. Monnereau, L.; Nieger, M.; Muller, T.; Bräse, S. *Adv. Funct. Mater.* **2014**, *24*, 1054–1058.
3. Plietzsch, O.; Schade, A.; Hafner, A.; Huuskonen, J.; Rissanen, K.; Nieger, M.; Muller, T.; Bräse, S. *Eur. J. Org. Chem.* **2013**, 283–299.
